# Oral Potentially Malignant Disorders and Oral Cancer in Saudi Arabia: An Epidemiological Review of the Literature

**DOI:** 10.3390/jcm13051376

**Published:** 2024-02-28

**Authors:** Khalid Aljohani, Ali Alqarni, Molly Harte, Rawia Alghamdi, Saja Alzahrani, Rui Albuquerque

**Affiliations:** 1 Department of Oral Diagnostic Sciences, Faculty of Dentistry, King Abdulaziz University, Jeddah 21589, Saudi Arabia; 2 Department of Oral & Maxillofacial Surgery and Diagnostic Sciences, Faculty of Dentistry, Taif University, Taif 21944, Saudi Arabia; aqarni@tu.edu.sa; 3 Department of Oral Medicine, Guy’s and St Thomas NHS Foundation Trust, Faculty of Dentistry, Oral Craniofacial Sciences, King’s College London, London SE1 8WA, UK; molly.harte@gstt.nhs.uk (M.H.); rui.albuquerque@gstt.nhs.uk (R.A.); 4 Independent Researcher, Jeddah 22335, Saudi Arabia; ralghamdi0592@stu.kau.edu.sa (R.A.); salzahrani0887@stu.kau.edu.sa (S.A.)

**Keywords:** oral potentially malignant disorders (OPMDs), oral pre-malignant disorders, oral malignancy, Kingdom of Saudi Arabia, squamous cell carcinoma, leukoplakia, erythroplakia, lichen planus, oral lichenoid lesions, oral submucous fibrosis

## Abstract

**Background:** Oral potentially malignant disorders (OPMDs) are a group of chronic oral mucosal diseases associated with an increased risk of malignant transformation. Multiple studies have investigated the prevalence of these conditions in multiple regions; however, there are limited data about the prevalence of OPMDs in the Kingdom of Saudi Arabia (KSA). This paper aims to review the prevalence of OPMDs in the KSA, to ensure better understanding of the population risk and propose a more standardised approach to the diagnosis and management of this group across the KSA. In addition, this review will discuss the prevalence of oral cancer in the KSA, considering independent risk factors for oral cancer development. **Methods:** Electronic databases including PubMed, Medline, Medscape, ScienceDirect, StatPearls, BMC Oral Health and the Cochrane Library were searched with the keywords “Oral Potentially Malignant Disorders”; “Saudi Arabia”; and “Oral Cancer”. Identified articles were reviewed independently by 2 reviewers against defined inclusion and exclusion criteria. **Results:** 16 studies were included in this review. The prevalence of OPMDs in KSA varies significantly depending on age, gender, social habits, background disease and dental status. **Conclusions:** This review highlights the need for up-to-date data on the prevalence, distribution, and characteristics of OPMDs in KSA. The diverse prevalence rates and distinct characteristics of various OPMDs emphasise the necessity for targeted preventive measures. As the data on OPMDs in KSA remains limited, future research efforts should prioritise the establishment of comprehensive epidemiological studies to inform effective public health interventions in this region.

## 1. Introduction

The term oral potentially malignant disorder (OPMD) encompasses several chronic oromucosal conditions associated with a risk of transformation to oral squamous cell carcinoma (OSCC) [1]. This group of disorders includes leukoplakia, erythroplakia, oral lichen planus and oral lichenoid lesions, and oral submucous fibrosis [1]. The diagnosis of these disorders can have profound implications on the overall health of those affected with an impact on the quality of life of both patients and their families [2,3]. The early diagnosis of these lesions is therefore essential to enable patient education, appropriate clinical monitoring, and risk reduction with respect to malignant transformation [4]. The epidemiology of OPMDs remains poorly explored in numerous global regions [2,3,5,6], including the Kingdom of Saudi Arabia (KSA), where standardised methods are lacking and comprehensive, up-to-date data on the prevalence, distribution, and characteristics of OPMDs among different subpopulations are scant [3,6]. This paucity of evidence poses a challenge to the development of effective prevention and intervention strategies for OPMDs in the KSA [6,7]. The prevalence of OPMDs globally varies significantly between populations, and therefore, population-based data may aid in the understanding of this variation.

The global epidemiology of OPMDs is complex and variable, as the prevalence, distribution, and characteristics of these disorders differ widely among different regions, populations, and subgroups, depending on the local risk factors, diagnostic methods, and surveillance systems [8]. Some of the most common risk factors for OPMDs are tobacco use, alcohol consumption, betel quid and areca nut chewing, and human papillomavirus infection, which may act synergistically or independently to induce genetic and epigenetic alterations in the oral mucosa, leading to dysplasia and malignancy [9,10]. The prevalence of OPMDs globally ranges from 0.02% to 25.9%, with higher rates reported in South and Southeast Asia, where the habit of betel quid and areca nut chewing is prevalent [11,12]. The malignant transformation rate of OPMDs is estimated to be between 0.13% and 36.4%, depending on the type, duration, and severity of the lesion as well as the presence of dysplasia on histopathological examination [13,14,15].

The oral health status of the population in the KSA is generally poor, with high prevalence of dental caries, periodontal diseases, and oral cancer [6,7]. However, the epidemiology of OPMDs in the KSA remains largely unknown, as there is a lack of standardised and comprehensive data on the prevalence, distribution, and characteristics of these disorders among different subpopulations, such as age groups, genders, regions, and socioeconomic statuses. This gap in knowledge hinders the development and implementation of effective prevention and intervention strategies for OPMDs in the KSA as well as the evaluation of their outcomes, and therefore, there is an urgent need for population-based studies to explore the epidemiology of OPMDs in the KSA, using reliable and valid diagnostic methods, and to identify the associated risk factors, clinical features, and molecular markers of these disorders. Such studies will provide valuable information for the planning and delivery of optimal oral health care services for the population in the KSA, as well as for the prevention and management of OPMDs and OSCC. The aim of this article is to conduct a comprehensive review of the existing literature, focusing specifically on the diagnosis of OPMDs and oral cancer and the prevalence of these disorders in the KSA.

## 2. Methodology

Searches were conducted on electronic databases such as PubMed, Medline, Medscape, ScienceDirect, StatPearls, BMC Oral Health, and the Cochrane Library with the Keywords “Oral Potentially Malignant disorders”; “Saudi Arabia”; and “Oral Cancer” (RA and SA). The initial search was then expanded by examining the reference sections of the articles found electronically. Additionally, a search was performed of the latest issues of prominent Oral Medicine journals (International Oral Health journal, Saudi medical journal, Journal of Oral and Maxillofacial Surgery, Medicine, and Pathology, The Saudi dental journal, Journal of Dentistry and Oral Biology, and Journal of oral pathology & medicine) to account for any publications not yet available in the electronic databases. After all that, potentially relevant articles were gathered, and a meticulous review of the article titles and abstracts was conducted. Before extracting data, reviewer calibration took place by instructing reviewers to extract information from the designated articles and assessing the similarity and consistency of the extracted data. During this step, two reviewers (RA and SA) independently evaluated the initial list of potentially relevant articles based on two specific eligibility criteria: 1. the study must have assessed prevalence data related to any oral potentially malignant disorders in any region of the KSA, and 2. the study must have presented original research findings that were meaningfully interpretable and published in English-language articles before and up to October 2023.

## 3. Results

**Figure 1:**
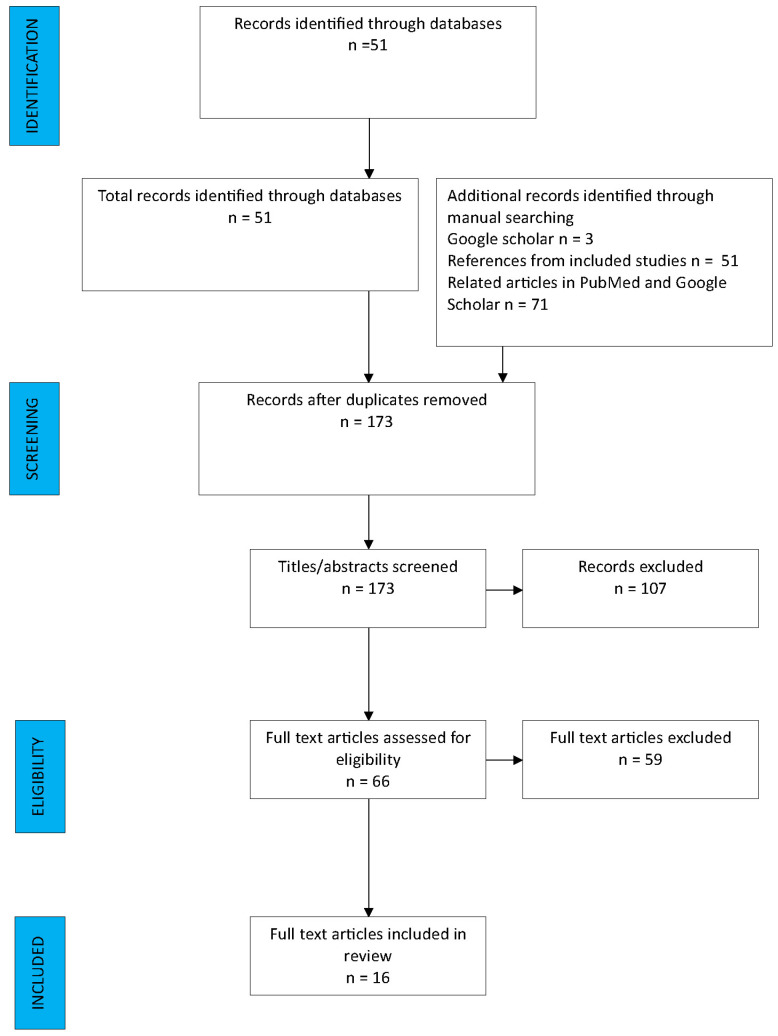
PRISMA flow diagram.

From the 180 articles initially selected, 164 studies were excluded following the removal of duplicates and the application of inclusion and exclusion criteria (Figure 1). A total number of 16 studies were included in this review [2,3,5,6,7,16,17,18,19,20,21,22,23,24,25,26].

The prevalence of OPMDs in the KSA was found to vary significantly depending on factors such as age, gender, social habits, systemic diseases, and dental status. Al-Attas (2014) [27] reported a greater prevalence of OPMDs among HIV-infected patients (16%) than in healthy controls (2%). Furthermore, the frequency of OPMDs exhibited disparities related to the geographical and contextual characteristics of the study (Table 1).

Table 1 summarises the findings of various studies on the prevalence of three types of oral lesions in Saudi Arabia: leukoplakia, lichen planus, and oral submucous fibrosis. Several factors contribute to the variation in the prevalence of leukoplakia, such as sample size, method, definition, and risk factors. The prevalence of lichen planus is influenced by the type, stage, and clinical form of the lesion as well as the diagnostic criteria and technique. The prevalence of oral submucous fibrosis is low due to the uncommon practice of betel quid chewing and tobacco use in Saudi Arabia and the likelihood of underdiagnosis and underreporting of the condition.

## 4. Leukoplakia

Leukoplakia describes white patches or plaques on the oral mucosa that cannot be characterised as any other definable lesion and that have a potential for malignant transformation. The prevalence of leukoplakia varies from 1.5% to 2.6% worldwide [28]; however, in the KSA, the reported prevalence ranges from 2.6 to 11.4% [2,3,19,23]. Leukoplakia is more prevalent in patients aged 60 years or older, and most published data globally report a male predilection [29], although in data from Riyadh city, oral leukoplakia was more commonly seen in women [30]. Leukoplakia can be classified as homogenous or non-homogenous based on the clinical appearance [31]. Homogenous leukoplakia presents as a white, well-defined plaque with a smooth or leathery surface [31]. Non-homogeneous leukoplakia presents as a white plaque with an irregular or nodular surface, sometimes mixed with red areas, termed erythroleukoplakia [1,31]. Leukoplakia can affect any site of the oral mucosa, but the floor of the mouth and the lateral borders of the tongue are considered high-risk sites for malignant transformation [32]. The treatment of leukoplakia involves surgical excision or laser ablation, but these modalities do not completely eliminate the risk of malignant transformation [33]. The risk of malignant transformation for leukoplakia in the KSA is not reported.

## 5. Oral Lichen Planus

Oral lichen planus (OLP) is a common chronic inflammatory condition that affects the oral mucosa in various patterns, ranging from the asymptomatic reticular type to the symptomatic erosive type [1]. The aetiology of OLP is not fully understood, but it is considered to be a cell-mediated immune response with possible genetic and environmental factors influencing development [34]. OLP is a potentially malignant disorder with transformation rates documented between 0.44 to 1.4% [35,36]. Salem has identified a 1.7% transformation rate of oral lichen planus when studying 4277 Saudi patients, from Gizan, Saudi Arabia [37]. Similar findings have been identified by Bandyopadhyay et al., who have identified 1,4% when studying retrospective patients from Bhubaneswar, Odisha [38]. As well as the mouth, lichen planus can also involve other mucocutaneous sites, such as the genitals, oesophagus, rectum, skin, scalp, and nails [39]. The prevalence of OLP varies from 0.5% to 2% worldwide [34]. In the KSA, the reported prevalence ranges from 0.35% to 11.08% according to different studies [2,3,5,6,17,23,24,26]. OLP is more prevalent in women than men and is seen with a higher frequency in patients older than 40 years at the time of diagnosis [40]. OLP can affect any oromucosal surface, but the most common sites are the buccal mucosae, lateral surfaces of the tongue, and the gingivae, where desquamative gingivitis may be seen [41,42]. The treatment of OLP is mainly aimed at controlling the symptoms and preventing complications [34]. First-line therapy is usually topical corticosteroids, and second-line therapy includes topical immunomodulators such as cyclosporine, tacrolimus, and retinoids [40,43].

## 6. Oral Lichenoid Lesions

Oral lichenoid lesions (OLLs) have a similar clinical and histopathological appearance to oral lichen planus (OLP) with a known antigenic trigger. OLL may develop as hypersensitivity reactions to medications or materials, such as antihypertensives, NSAIDs, penicillin, amalgam, and/or gold [43]. No data exist on the prevalence of OLL in the KSA.

## 7. Graft-versus-Host Disease

Graft-versus-host disease (GvHD) occurs in patients post-haemopoetic stem cell transplant, and oral lichenoid lesions may be encountered as part of the mucocutaneous presentation of GvHD. The incidence of GvHD ranges between 15% and 50%, and the risk increases with the age of the transplant recipient [44]. In the KSA, Aboalela et al. reported oral GvHD in 30.6% of patients, identifying weight loss was more prevalent among oral GvHD, and oral mucositis was linked to significant weight loss [45]. Patients with OLL associated with GvHD undergo similar treatment as for OLP, but often require closer monitoring due to both the potentially malignant nature of OLL in combination with the risk of secondary malignancies arising as a complication of GvHD [46].

## 8. Oral Submucous Fibrosis

Oral submucous fibrosis (OSMF) is a chronic disorder, which can affect the oral mucosa, pharynx, and the upper two-thirds of the oesophagus [1]. The main aetiological factor is areca nut chewing [47]. Al-Attas et al. (2014) [2] reported a similar trigger in the Jeddah population, Saudi Arabia. The malignant transformation risk of OSMF has been reported to range from 1.2% to 23% [48,49]. The most common intraoral sites of OSMF are the lips, buccal mucosa, retromolar area, and/or soft palate [50,51,52,53]. The prevalence of OSMF is around 2–5%, with a higher incidence in individuals less than 20 years old [54]. In the KSA, OSMF accounts for 0.08–0.5% of the reported oral lesions [2,6]. The initial signs of OSMF are erythematous lesions, followed by mucosal pallor [55]. The most characteristic clinical feature is the presence of fibrotic bands beneath the atrophic epithelium [55]. The diagnosis of OSMF is based on the history of areca nut chewing, clinical manifestations, and histopathological findings [55]. The diagnostic criteria include at least one of the following: palpable fibrous bands, mucosal texture that feels tough and leathery, and blanching of mucosa along with histopathologic features consistent with OSMF: atrophic epithelium with loss of rete ridges and juxta-epithelial hyalinisation of lamina propria [49,55]. The management of OSMF begins with cessation of the chewing habit. In advanced stages, various therapeutic modalities have been attempted, such as topical and systemic corticosteroids, hyaluronic acid, interferon-γ, supplementation of vitamins and nutrients, repeated dilatation with physical devices, and surgery [56,57].

## 9. Erythroplakia

Oral erythroplakia is a red lesion of the oral mucosa that cannot be characterised as any other definable lesion [1]. It is less common than leukoplakia, with a prevalence of 0.02% to 0.1% in adults [58]. The reported global mean prevalence of oral erythroplakia has been reported as 0.11% (ranging from 0.01 to 0.21%). Malignant transformation rates of erythroplakia are high, ranging from 14% to 85% [59,60]. In the KSA, the reported prevalence is higher than the global prevalence at 0.2% [2]. The diagnostic process is the same as for leukoplakia. Unlike leukoplakia, which can be closely monitored for years as an alternative to surgical treatment, biopsy and surgical excision is the recommended approach for managing erythroplakia given its higher risk of malignant transformation [61].

## 10. Risk Factors for Oral Cancer: Social and Cultural Habits in Saudi Arabia

Smokeless tobacco (ST) is unburned tobacco, which is powdered and placed in the buccal or labial vestibules [62]. Its use has been associated with adverse effects on oral and dental health, including tooth discoloration, tooth loss, gingival and periodontal disease, mucosal changes, pre- and potentially malignant lesions, and oral squamous cell carcinoma [2]. In the southwestern regions of the KSA, including the Jazan province, Shammah is a frequently used form of ST [61]. Khat is another form of ST used frequently by Yemeni immigrants in the KSA [22,24]. Khat is a stimulant plant that is also chewed or brewed as tea. Both of these plants are widely used in Yemen and some parts of Saudi Arabia, especially in the Jazan region, which borders Yemen [61]. Shammah use is associated with an increased risk of oral potentially malignant disorders (OPMDs) and oral cancer [61]. Shammah can cause chronic inflammation, DNA damage, and oxidative stress in the oral mucosa, which may lead to malignant transformation [61]. Khat use may also have some carcinogenic effects, as it contains substances that can interfere with cell cycle regulation and apoptosis [63]. Therefore, it is possible that the high prevalence of Shammah and Kat use among Yemeni immigrants and residents of the Jazan region may account for the higher incidence of OPMDs and oral cancers in these regions.

A strong relationship between ST and oral mucosal change has been reported in several studies in the KSA [2,24,62,64]. Tandon et al. (1995) [65] found a positive history of Shammah use in 98.7% of patients presenting with oral mucosal changes, with lesions seen across various oromucosal sites including the tongue, floor of mouth, lower lip, labial vestibule, cheek, buccal vestibule, alveolar mucosa, and gingival mucosa. The prevalence of leukoplakia in the Jazan region ranged from 11.4% to 68%, with 99% of patients in this study reporting regular Shammah use. Also demonstrated in this study was a strong association between the site of ST placement and the location of the oral lesion [66].

Smoking is considered socially acceptable in the KSA, unlike alcohol consumption and drugs where both are prohibited as well. Smoking is a well-documented and understood risk factor for the development of oral premalignant diseases and oral cancers. Overall, 12.1% to 15.3% of Saudis reported that they currently smoke tobacco. This prevalence was 23.7–28.9% among males and 1.5–2% among females [67]. In fact, the most common form of tobacco usage in the KSA is cigarette smoking, reporting 65.6%, followed by shisha with 38.1%. OPMDs lesions associated with smoking habits account for 10.5% [2].

Alcohol is known to be a risk factor for oral cancer, particularly when consumption of alcohol is in combination with smoking. In the KSA, there are inadequate data relating to alcohol consumption, but reported cases suggested a percentage between 10.6 and 16.10 of the population. This low percentage could be due to alcohol manufacturing, sale, consumption, and possession are known to be illegal based on the law and regulations of the country [68,69].

## 11. Other Risk Factors for Oral Cancer

Human papilloma virus (HPV) commonly causes benign oral squamous papillomas but high-risk strains have also been demonstrated in dysplastic lesions and is a separate risk factor for oropharyngeal cancer [70]. In the US, HPV is the leading cause of oropharyngeal carcinoma and accounted for 70% of all cases [71]. HPV-related oropharyngeal cancers in the KSA are reported as 0.06 in 100,000 male patients and 0.05 in 100,000 female patients, respectively [72].

## 12. Oral Cancer in Saudi Arabia

Oral potentially malignant disorders (OPMDs) are lesions with a risk of malignant transformation. For all OPMDs described above, this risk is specifically associated with the development of oral squamous cell carcinoma (SCC), which is the most common oral malignancy worldwide [73]. Although not arising from OPMDs, other oral malignancies have been reported in the literature in the KSA including salivary gland malignancies—adenoid cystic carcinoma, mucoepidermoid carcinoma—lymphoma, melanoma, sarcoma, and metastatic tumours from other sites [22,24,74]. The incidence of oral malignancies as reported in the literature from the KSA ranged from 0.5 to 1.8 per 100,000 population in different studies [6,17,18,19,21,22,23,24] (Table 2). The most frequently encountered oral malignancy in the KSA was SCC, in keeping with the global data (reference). The mortality rate of oral malignancies reported in Saudi residents ranged from 0.2 to 0.8 per 100,000 population in different studies [16,22,23,24,74]. Regional variation in oral cancer incidence was evident, as Jazan had the highest age-standardised rate and Hail had the lowest. Oral cancer incidence increased with age [72].

As well as the variability dependent on the malignancy type, incidence and mortality also varied according to the age, gender, smoking status, and patient past medical history. Idris et al. (2016) [22] found that the incidence of oral malignancy was higher among older patients (>60 years) than younger patients (<40 years) and among males than females. Mani (1985) [23] found that the mortality of oral malignancy was higher among HIV-infected patients than healthy controls. Al-Mohaya et al. (2009) [16] found that the incidence of oral malignancy was higher among renal transplant patients than healthy controls. Saudi studies found a higher incidence of oral malignancy in smokers and smokeless tobacco users [2,66].

As regards to regional variation, Idris et al. (2016) [22] found that the incidence of oral malignancy was higher in the Jazan province than in other regions of Saudi Arabia. Mani (1985) reported higher mortality associated with oral malignancy in Riyadh city than in other cities in Saudi Arabia [23]. Al Wayli et al. (2016) found that the incidence of oral malignancy was higher in female patients visiting a tertiary dental health centre in the Riyadh region than among female patients visiting a primary health care centre in the Riyadh region [26]. However, a referral bias should be considered as the Riyadh region is home to the King Faisal Specialist Hospital & Research Centre, the primary centre for the treatment of oral cancer in the KSA, with remaining treatment taking place across secondary hospitals—military, university, and ministry of health centres.

## 13. Discussion

Oral potentially malignant disorders represent an important and significant health problem and are therefore a widely researched area. High quality data exist relating to the prevalence, diagnostic techniques, and management of OPMDs for numerous geographical regions [10,75,76], but equivalent evidence relating to OPMDs in the KSA is lacking. The lack of such data poses a huge challenge to the development of effective prevention and intervention strategies for patients with OPMDs in the region. Without a clear understanding of the prevalence rates of these disorders in the KSA, targeted health care policies cannot be implemented, and resources cannot be appropriately allocated to those geographical regions which are most in need. Early diagnosis of OPMDs not only enables patient education and intervention but is also implemented in risk reduction as regards to the malignant transformation of these lesions [4]. Screening programmes have been shown to be an effective tool for early identification of OPMDs and oral malignancies [77], but can only be put into place with the backing of clear epidemiological data.

In our review article, we focus on peer-reviewed articles to ensure scientific rigor. ensuring the information identified has undergone rigorous evaluation by external reviewers, providing reliable information enhancing the article’s credibility and ensuring methodological soundness [78]. Authors have searched a posteriori relevant websites including the World Health Organisation, Saudi Health Council, and Global Cancer Registry; however, the data provided focused on cancer with no obvious data or studies focusing on oral potentially malignant disorders, which would comply with our inclusion criteria. The prevalence of OPMDs is influenced by a variety of factors such as age, gender, social habits, and systemic disease, and regional data suggest a significant variance in the prevalence of OPMDs globally, with the highest prevalence reported in Asia [79]. Social and cultural habits are frequently implicated in this geographical prevalence variation, with the high prevalence of OPMDs in Asia likely related to areca nut chewing. In Saudi Arabia, Shammah is a commonly used as a smokeless tobacco preparation that comprises tobacco, lime, ash, black petter, oils, and flavourings. It is held in the mouth and has been linked to the development of oral cancer as well as oral premalignant lesions [80]. When considering the prevention of potentially malignant and malignant disorders in the KSA, it is essential to understand the cultural and social habits of the population and the implication these habits may have on the development of such lesions, in order to better educate both the public and health care professionals about this risk. Various oral habits, such as Khat and Shammah chewing among Yemenis, Toombak dipping among Sudanese, and cigarette and shisha smoking among Egyptians, are prevalent among non-Saudi nationals residing in Saudi Arabia [79,81]. These habits may increase the likelihood of OPMD and oral cancer development.

The approximate risk of progression to malignancy is specific to the exact diagnosis; for example, oral submucous fibrosis has a reported transformation rate as high as 23% [82], whereas the risk associated with lichen planus is much lower, reported as up to 2.28% [83]. This clearly necessitates different approaches when it comes to intervention and monitoring of these lesions and an agreed standardised approach to management based on national data may reduce regional health inequalities.

A recent work suggests that other lesions such as vulvar lichen sclerosus may indirectly, but significantly, increase the risk of oropharyngeal tumours [84]. Perhaps most importantly, the lack of evidence on OPMDs in the KSA impedes international comparison and collaboration. Robust epidemiological data contribute not only to national health policies but also facilitate collaboration with global health organisations, research institutions, and improve understanding of best practices both in diagnosis and management of these lesions. The global variation in the prevalence of OPMDs emphasises the need for a collaborative, international effort to address these disorders comprehensively [85].

In conclusion, this review underscores the need for standardised methods and up-to-date data on the prevalence, distribution, and characteristics of OPMDs in the KSA. The diverse prevalence rates and distinct characteristics of various OPMDs emphasise the necessity for targeted preventive measures. As the data on OPMDs in the KSA remain limited, future research efforts should prioritise the establishment of comprehensive epidemiological studies to inform effective public health interventions. This review contributes to the existing knowledge base, urging stakeholders to address the gaps in research and work collaboratively towards enhancing the understanding and management of oral potentially malignant disorders in the KSA.

## 14. Recommendations

Based on this extensive review of the existing literature relating to OPMDs in the KSA, we make the following recommendations to healthcare professionals in the KSA involved in the management of patients with oral potentially malignant disorders to improve outcomes in this group.

1. A thorough clinical examination, including extra-oral head and neck examination and intra-oral soft tissue examination, should be carried out by all primary care dentists when seeing any patient.
2. Patients with known oral potentially malignant disorders should be followed up longitudinally with clinical photographs to aid accurate documentation and monitoring.
3. A list of high-risk clinical features and social factors associated with oral potentially malignant disorders and oral malignancy should be established specific to the population of the KSA.

## Figures and Tables

**Table 1:** The prevalence of OPMDs in the published papers in the KSA.

Oral Lesion	Prevalence (%)	Author, Year
Leukoplakia	0.85%	Ali et al., 2013 [19]
	3.17%	Al Jabab et al., 2015 [3]
	1.9%	Mani, 1985 [23]
	2.3%	Al-Attas et al., 2014 [2]
	2.8%	Alanazi Y et al., 2016 [17]
Lichen planus	0.35%	Al-mobeeriek and Aldosari, 2009 [5]
	6%	Al Jabab et al., 2015 [3]
	11.08%	Al Wayli et al., 2016 [26]
	4.4%	Alhindi et al., 2019 [6]
	1.5%	Saleh et al., 2017 [24]
	3.36%	Alblowi and Binmadi, 2018 [18]
	0.6%	Mani, 1985 [23]
	0.4%	Al-Attas et al., 2014 [2]
	7.1%	Alanazi et al., 2017 [17]
Oral submucous fibrosis	0.5%	Al-Attas et al., 2014 [2]
	0.08%	Alhindi et al., 2019 [6]
Erythroplakia	0.2%	Al-Attas et al., 2014 [2]

**Table 2:** Types and anatomical distribution of oral malignancies according to the region to which the patient belongs.

Author	Year	Region/City	Number of Patients	Type of Tumour	Location
Mani [23]	1985	Riyadh	674	Squamous cell carcinoma	Tongue
Ali et al. [19]	2013	Eastern province	3150	Hodgkin’s lymphoma Non-Hodgkin’s lymphomas Papillary carcinoma of thyroid gland Squamous cell carcinoma Basal cell carcinoma Undifferentiated carcinoma Adenoid cystic carcinoma Mucoepidermoid carcinoma Cutaneous melanoma	Head and neck region
Idris et al. [22]	2016	Jazan	714	Squamous cell carcinoma Verrucous carcinoma Oral precancer/epithelial dysplasia Ameloblastic carcinoma Salivary gland malignancy Sarcoma	Tongue Floor of mouth Bucco-alveolar mucosa
Saleh et al. [24]	2017	Jazan	714	Squamous cell carcinoma Verrucous carcinoma Mucoepidermoid carcinoma Adenoid cystic carcinoma adenocarcinoma Ameloblastic carcinoma Small round cell sarcoma Alveolar soft part sarcoma Ewing’s sarcoma Undifferentiated tumour Metastatic tumour	Tongue Floor of mouth Bucco-alveolar mucosa
Bello and Qannam [21]	2022	Riyadh	624	Squamous cell carcinoma Non-Hodgkin lymphoma Rhabdomyosarcoma Malignant fibrous histiocytoma Melanoma Verrucous carcinoma Malignant peripheral nerve sheath tumour Kaposi sarcoma	Gingiva Alveolar ridge
